# Cognitive profile differences in primary progressive aphasia between Spanish and English speakers

**DOI:** 10.1002/alz70857_099449

**Published:** 2025-12-24

**Authors:** Lucia Fernandez‐Romero, James Carrick, Ramon Landin‐Romero, David Foxe, Miguel Yus Fuertes, Alberto Marcos Dolado, Jordi A Matias‐Guiu, Olivier Piguet

**Affiliations:** ^1^ Hospital Clinico San Carlos, Madrid, Madrid, Spain; ^2^ The University of Sydney, Brain and Mind Centre, Sydney, NSW, Australia; ^3^ Hospital Clinico Universitario San Carlos, Madrid, Madrid, Spain

## Abstract

**Background:**

Primary progressive aphasia (PPA) comprises neurodegenerative syndromes characterized by progressive language impairment. Cultural and linguistic differences may determine the presentation of PPA variants, influencing both language deficits and other cognitive domains. This study investigates cognitive profiles in English‐ and Spanish‐speaking participants with PPA from Australia and Spain, exploring cross‐cultural variations.

**Method:**

This cross‐sectional study included 461 participants: 215 from Australia (37 nonfluent PPA (nfvPPA), 49 semantic PPA (svPPA), 45 logopenic (lvPPA) and 84 healthy controls (HC)) and 246 from Spain (64 nfvPPA, 31 vsPPA, 67 lvPPA, and 84 HC). Diagnoses were made according to established criteria, with comprehensive language assessments and neuroimaging. Cognitive performance was assessed using the ACE‐III and additional cognitive tests. Cognitive performance was compared across clinical groups and cohorts. Structural brain MRI of a subgroup of patients were also compared across cohorts.

**Result:**

Cognitive performance differed significantly across PPA variants and HC in both cohorts. Spanish PPA groups generally scored lower than Australian PPA groups on ACE‐III total and specific subdomains, particularly attention, memory, and visuospatial abilities. Visuospatial impairment was more pronounced in the Spanish nfvPPA group. Cortical thickness patterns were consistent across cohorts, with no evidence of greater atrophy in the Spanish cohort was found.

**Conclusion:**

These findings revealed consistent impairments in non‐language cognitive domains across Australian and Spanish cohorts with PPA. Spanish patients exhibited more generalized cognitive deficits compared to Australians, despite similar demographic, functional, and neuroimaging profiles. These findings suggest that non‐linguistic factors, such as cultural factors, cognitive reserve, or resilience, may influence the presentation of PPA.

